# Large datasets of water vapor sorption, mass diffusion immersed in water, hygroscopic expansion and mechanical properties of flax fibre/shape memory epoxy hygromorph composites

**DOI:** 10.1016/j.dib.2022.108367

**Published:** 2022-06-10

**Authors:** Qinyu Li, Rujie Sun, Antoine Le Duigou, Jianglong Guo, Jonathan Rossiter, Liwu Li, Jinsong Leng, Fabrizio Scarpa

**Affiliations:** a Bristol Composites Institute, University of Bristol, BS8 1TR Bristol, UK; b Department of Materials, Department of Bioengineering and Institute of Biomedical Engineering, Imperial College London, London, UK; c Polymer and Composites, Université Bretagne Sud, UMR CNRS 6027, IRDL, F-56100 Lorient, France; d School of Science, Harbin Institute of Technology (Shenzhen), Shenzhen, People's Republic of China; e SoftLab, Bristol Robotics Laboratory, University of Bristol, Bristol, UK; f Department of Astronautical Science and Mechanics, Harbin Institute of Technology (HIT), P.O. Box 301, No. 92 West Dazhi Street, Harbin 150001, P. R. China; g National Key Laboratory of Science and Technology On Advanced Composites in Special Environments, Harbin Institute of Technology (HIT), No.2 Yikuang Street, P.O. Box 3011, Harbin 150080, P. R. China

**Keywords:** Natural fibres, Biocomposite, Moisture absorption, Hygroscopic expansion, Mechanical testing

## Abstract

This data article presents four experimental sets of results related to flax fibre composites with epoxy shape memory polymer matrix: water vapor absorption, mass diffusion immersed in water, hygroscopic expansion, mechanical properties. The water vapor absorption tests are described in raw data related to four types of laminates with weights measured at different relative humidity (0%, 9%, 33%, 44%,75%, 85% and 100%). The mass diffusion experiments are related to weights of immersed samples over time. The unidirectional composite hygroscopic expansion is also measured along the fibre longitude and transverse directions. The mechanical properties of flax composite at various temperatures (20°C, 40°C, 60°C, 80°C and 100°C) and humidity environments (50% and immersed) are also described. Load-displacement diagrams of the hygromorph composites are converted into stress-strain diagrams via a compliance calibration, from which the tensile moduli are extracted. The data presented in this article can provide a benchmark for the development of new models, or for the determination of other properties via post processing. The detailed interpretation of the data can be found in [1]. The data is available in the Mendeley Data repository at [2].

## Specifications Table

**Table:** 

Subject	Ceramics and Composites
Specific subject area	Flax fibres composite
Type of data	Table (comma separated values)
How the data were acquired	Vapor water sorption was acquired in the following way: • The samples used were made from flax fibre composites with 4 different stacking sequences [90_8_], [0_1_90_7_], [0_2_,90_6_] and [0_3_90_5_]. The quantity [0_m_90_n_] indicates m laminas along the fibre longitudinal direction and n laminas along the transverse one. • The relative humidity (RH) was generated via saturated solutions of potassium hydroxide (KOH), magnesium chloride (MgCl_2_), potassium carbonate (K_2_CO_3_), sodium chloride (NaCl), potassium chloride (KCl) and water. Those salts created RH conditions of 9%, 33%, 44%,75%, 85% and 100%, respectively. • Samples were stored for more than 48 hours in the specific relative humidity conditions. The samples were weighted with a balance with an accuracy of 10^−3^ g. Mass diffusion was measured as follows: • Four (4) different laminates of flax/epoxy composites were used. The architectures had the following stacking sequences: [90_8_], [0_1_90_7_], [0_2_,90_6_] and [0_3_90_5_]. • The samples for absorption were initially stored at 50% RH and then immersed in water. The weights of the samples over time have been measured using a balance with an accuracy of 10^−3^ g. • The saturated samples have been dried at 50% RH, and the weights resulting in desorption measured over time in the same way followed for absorption. The hygroscopic expansion has been measured in the following manner: • Samples consisted in unidirectional laminates [90_8_]. • The specimens were stored at the above-mentioned RH conditions (9%, 33%, 44%,75%, 85% and 100%). • The dimensions of the samples were measured using a Mitutoyo micrometre.
	The mechanical properties were measured in the following way: • The samples considered were unidirectional laminates [0_8_] and [90_8_]. • Samples were tested according to ISO 527-4 standards, using a Shimadzu universal testing machine (cell load 5 kN) with a crosshead speed of 1 mm/min. • Samples at RH 50% and in immersed conditions were wrapped with polymer films to prevent the loss of moisture during the tests. • A heating chamber (TCE-N300A Shimadzu, UK) with a Shimadzu universal testing machine to control the setting of the temperature prior test and thermocouples (621-2170 from RS components, UK) were also used to verify the temperature close to the samples.
Data format	Raw (weights and dimensions of samples, time temperature), filtered (force-displacement diagrams) and analysed (moisture content, diffusion parameters, hygroscopic expansion, stress-strain diagrams, tensile modulus).
Description of data collection	• Samples at various RH conditions were weighted by a balance with an accuracy of 10^−3^ g. • The dimensions of samples were measured by a Mitutoyo micrometre. • Force-displacement diagrams of tensile condition were tested by a Shimadzu universal testing machine (cell load 5 kN)
Data source location	Institution: University of Bristol City/Town/Region: Bristol Country: UK Latitude and longitude (and GPS coordinates, if possible) for collected samples/data: 51.4584° N, 2.6030° W
Data accessibility	DOI: 10.17632/t7fr46vjcs.1 at [2]
Related research article	Q. Li, R. Sun, A. Le Duigou, J. Guo, J. Rossiter, L. Liu, J. Leng, F. Scarpa, Programmable and reconfigurable hygro-thermo morphing materials with multifunctional shape transformation, Appl. Mater. Today 27, 101414. https://doi.org/10.1016/j.apmt.2022.101414

## Value of the Data

• Natural fibres composites possess interesting mechanical properties, are relatively low-cost and environmentally friendly. These bio-based composites are moisture sensitive. It is therefore critical to understand their transport properties (water vapor sorption, mass diffusion), their hygroscopic expansion and the variation of the tensile Young's modulus at different thermal and humidity conditions. The knowledge of those characteristics can be used to design similar types of biocomposites, and/or benchmark classes of materials. Detailed datasets related to flax composites allow to generating high fidelity simulations of those composites and in general predicting and designing advanced biobased materials.
• Researchers who study natural fibres composites properties affected by the humidity would benefit from the access to these data that allow to understand the moisture content of these biocomposites at different humidity levels, the hygroscopic expansion and variation of mechanical properties.
• The datasets here is related to four experiments and quantities (sorption of moisture content, diffusivity, hygroscopic expansion ratio and Young's modulus). These materials metrics can be directly used in analytical and numerical models, also to benchmark other types of natural fibres composites. To the best of our knowledge, similar types and quality of data for biobased composites and hygromorph systems are not completely available in open literature.

## 1 Data Description

The file names are related to: 1-Water Vapor Sorption, 2-Mass diffusion, 3- Hygroscopic Expansion and 4- Tensile Property.

1- **Water Vapor Sorption**

The water vapor sorption file contains two files: the original weights of the samples at various RH conditions (named ‘weight’) and the moisture content at different RH percentages (‘moisture content’).

The ‘weight’ file is structured in the following way:

• Column 1: Specimen index; ‘8-1’ means the [90_8_] laminate number 1 sample.
• Column 2 to Column 12: weights of samples at 9%, 33%, 44%, 75%, 85%, 100%, 85%, 75%, 44%, 33%, 9%. The unit is mg.

The ‘moisture content’ file has the following data structure:

• Column 1: Specimen index; ‘8-1’ means the [90_8_] laminate number 1 sample.
• Column 2 to Column 12: moisture content of samples at 9%, 33%, 44%, 75%, 85%, 100%, 85%, 75%, 44%, 33%, 9%. The unit is %.

2- **Mass Diffusion**

The mass diffusion section contains six files: three of them are referred to the original weights of the immersed samples over time for the 1^st^ and 10^th^ cycle absorptions and the 10^th^ cycle desorption, respectively. The files are named ‘1st_absorb_weight’, ‘10th_ absorb_weight’, and ’10th_desorb_weight’. The other three files contain data about the moisture content over time (‘analysis_1stcycle_absorb_massdiffusion’, ‘analysis_10thcycle_absorb_massdiffusion’ and ‘analysis_10thcycle_desorb_massdiffusion’).

The ‘_weight’ files data structure is the following:

• Column 1: Specimen index; ‘8-1’ means the [90_8_] laminate number 1 sample.
• Column 2 to end Column: weights of samples at over time. The unit is mg.

The ‘_massdiffusion’ files have the following data structure:

• Column 1: Specimen index; ‘8-1’ means the [90_8_] laminate number 1 sample.
• Column 2 to end Column: moisture content of samples at over time. The unit is %.

3- **Hygroscopic Expansion**

The hygroscopic expansion section contains two files. One is related to the 7 samples original weights, lengths, and widths when they absorb moisture (named ‘length and width’). The other is referred to the hygroscopic expansion with different moisture contents (named ‘longitude and transverse expansion’).

The data structure of the ‘length and width’ files is the following:

• Column 1 to 5 are: weights of samples (g), length of two positions and width of two positions (mm).

For ‘longitude and transverse expansion’ files the data structure is:

• Column 1: moisture content (%)
• Column 2: longitude expansion (%)
• Column 3: transverse expansion (%)

4- **Tensile Properties**

The tensile properties section contains three parts: the ‘force-displacement’ and the ‘strain-stress’ folders, and the ‘Tensile modulus’ file.

The ‘force-displacement’ folder contains data related to 100 tests. The files have names like ‘L-20-dry-1’, meaning (L)ongitude direction, 20°C temperature, 50%RH (dry) and 1^st^ tested sample. Other files have the name structure like ‘T-100-wet-2’ i.e., (T)ransverse direction, 100°C temperature, immersed condition (wet) and 2^nd^ tested sample. The data are structured as follows:

• Column 1: force (N)
• Column 2: displacement (mm)

In the ‘strain-stress’ folder, the way the names of the files have been attributed follows the same rationale of the ‘force-displacement’ folder. The data in the columns has a structure like:

• Column 1: strain
• Column 2: stress (MPa)

The structure of the ‘Tensile modulus’ file data is:

• Column 1: Specimen Index (1-100)
• Column 2: direction: longitude or transverse
• Column 3: temperature (°C)
• Column 4: Humidity: 50% or Immersed
• Column 5: Tensile modulus (GPa)

## 2 Experimental Design, Materials and Methods

• Material supplier and processing

Unidirectional flax fibre tapes (50 g/m^2^) have been supplied by Nat-up, France. The flax fibres are fixed by tape on their boundaries and cut into a 250mm*250mm size. Stacks of shape memory epoxy films [3] and unidirectional flax-fibre tapes have been cured in autoclave at 0.69MPa pressure with the following temperature profile: 80°C for 3 hours, 100°C for 3 hours and 150°C for 5 hours. Overall, we have used an 8-layer flax tape. The flax fibre composites have 4 different bilayer architectures: [90_8_], [0_1_90_7_], [0_2_,90_6_] and [0_3_90_5_].

• Water vapor sorption

Samples have been stored in a reference condition within a vacuum oven at 60 °C, assumed to be close to 0% RH humidity [4]. During the experiments the samples have been stored in boxes with relative humidity controlled by saturated solutions of potassium hydroxide (KOH), magnesium chloride (MgCl_2_), potassium carbonate (K_2_CO_3_), sodium chloride (NaCl), potassium chloride (KCl). The salts allowed to reach RH values of 9%, 33%, 44%,75%, 85% and 100%, respectively. The samples were then weighed using a balance with an accuracy of 10^−3^ g after reaching saturation, i.e., when the weight was stabilized. A period of 48 hours has been considered acceptable to reach the stabilization of the weight.

• Mass diffusion

Samples have been initially stored at 50% RH and 23°C to attain the reference state. The materials were then immersed in deionized water at room temperature. During immersion, the samples have been periodically removed to be weighed using a balance with an accuracy of 10^−3^ g, and then characterized:

(1) $$C_{\left(t\right)}=\frac{W_{t}\;-W_{0}}{W_{0}}\cdot 100$$

In (1), $W_{t}$ and $W_{0}$ are the weight of the sample at a time *t* after water exposure and the weight of the dry material before immersion (for RH = 50% and T = 23°C).

• Hygroscopic expansion

Volumetric measurements [4] have been performed with a Mitutoyo micrometre IP65 (10^−3^mm). The gravimetric analyses have been made using a Fischer Scientific PAS214C balance (10^−3^ g). The coefficient of hygroscopic expansion *(β)* has been determined as the slope of the hygroscopic expansion over the moisture content*.*

(2) $$\beta_{\left(c\right)}=\frac{\;L_{c}-L_{0}}{L_{0}}\cdot 100$$

In (2), $L_{c}$ and $L_{0}$ are the sizes of the sample at a time t after water exposure and before immersion, respectively*.*

• Elastic properties

Tensile properties of dry (50% RH) and wet (immersed) unidirectional biocomposites with flax fibres orientations at 0° *(E_L_)* and 90° *(E_T_)* have been measured according to the ISO 527-4 standards by using a Shimadzu universal testing machine (cell load 5 kN) at controlled temperature (23°C) with a crosshead speed of 1 mm/min. The samples had the following dimensions: *t_0_*_°_=0.56 mm and width *w_0°_* = 15 mm; *t_90°_*=0.56mm and *w_90°_*= 25 mm. The tests were performed on samples that reached the saturation state. The samples were covered with saran wrap to prevent the loss of moisture. A heating chamber (TCE-N300A, Shimadzu, UK) to control the setting temperature and thermocouples (621-2170 from RS components, UK) were also used to verify the temperature close to the samples. An axial extensometer (560S Shimadzu, UK) with a nominal length of 25 mm *(L_0_)* was used to measure the strain.

## Ethics Statements

This work has not involved any use of human subjects and animal experiments.

## CRediT Author Statement

**Qinyu Li:** Conceptualization, Methodology, Software, Investigation, Writing – Original Draft, Visualization; **Rujie Sun:** Conceptualization, Validation, Formal analysis, Writing – review & editing; **Antoine Le Duigou:** Resources, Writing – review & editing, Supervision; **Jianglong Guo:** Conceptualization; **Jonathan Rossiter:** Conceptualization, Writing – review & editing; **Liwu Liu:** Resources, Writing – review & editing; **Jinsong Leng:** Resources, Writing – review & editing, Supervision; **Fabrizio Scarpa:** Resources, Writing – review & editing, Supervision, Project administration, Funding acquisition.

## Declaration of Competing Interest

The authors declare that they have no known competing financial interests or personal relationships that could have appeared to influence the work reported in this paper.

## Data Availability

Large datasets of water vapor sorption, mass diffusion immersed in water, hygroscopic expansion and mechanical properties of flax fibre/shape memory epoxy hygromorph composites (Original data) (Mendeley Data).
